# Divergent selection on flowering phenology but not on floral morphology between two closely related orchids

**DOI:** 10.1002/ece3.6312

**Published:** 2020-05-06

**Authors:** Elodie Chapurlat, Iris Le Roncé, Jon Ågren, Nina Sletvold

**Affiliations:** ^1^ Plant Ecology and Evolution Department of Ecology and Genetics Evolutionary Biology Centre Uppsala University Uppsala Sweden; ^2^ Master BioSciences École Normale Supérieure de Lyon Université Claude Bernard Lyon 1 Université de Lyon Lyon France

**Keywords:** divergent selection, flowering phenology, *Gymnadenia*, phenological isolation, plant–pollinator interactions, reproductive barriers, species divergence

## Abstract

Closely related species often differ in traits that influence reproductive success, suggesting that divergent selection on such traits contribute to the maintenance of species boundaries. G*ymnadenia conopsea* ss. and *Gymnadenia densiflora* are two closely related, perennial orchid species that differ in (a) floral traits important for pollination, including flowering phenology, floral display, and spur length, and (b) dominant pollinators. If plant–pollinator interactions contribute to the maintenance of trait differences between these two taxa, we expect current divergent selection on flowering phenology and floral morphology between the two species. We quantified phenotypic selection via female fitness in one year on flowering start, three floral display traits (plant height, number of flowers, and corolla size) and spur length, in six populations of *G. conopsea* s.s. and in four populations of *G. densiflora.* There was indication of divergent selection on flowering start in the expected direction, with selection for earlier flowering in two populations of the early‐flowering *G. conopsea* s.s. and for later flowering in one population of the late‐flowering *G. densiflora*. No divergent selection on floral morphology was detected, and there was no significant stabilizing selection on any trait in the two species. The results suggest ongoing adaptive differentiation of flowering phenology, strengthening this premating reproductive barrier between the two species. *Synthesis*: This study is among the first to test whether divergent selection on floral traits contribute to the maintenance of species differences between closely related plants. Phenological isolation confers a substantial potential for reproductive isolation, and divergent selection on flowering time can thus greatly influence reproductive isolation and adaptive differentiation.

## Graphical abstract

We document divergent selection on flowering phenology in two closely related orchids. The results suggest ongoing adaptive differentiation, where selection is strengthening divergence in flowering phenology, an important premating reproductive barrier between the two species.

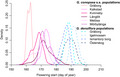

## 1 INTRODUCTION

In angiosperms, flowering time and flower morphology critically influence mating patterns because of their effects on pollen transfer. Timing of flowering determines which pollinators can visit the flowers (Elzinga et al., 2007) and the shape, color, scent, and size of flowers and inflorescences are important traits for attracting pollinators and/or for the efficiency of pollination (Ida & Kudo, 2010; Jersáková, Jürgens, Šmilauer, & Johnson, 2012; Raguso, 2008; Trunschke, Sletvold, & Ågren, 2019). Differentiation in floral traits between taxa may thus play an important role in reducing interspecific pollen transfer and contribute to reproductive isolation, either through phenological isolation (premating barrier caused by differences in flowering time; e.g., Kudo, 2006; Nuismer & Cunningham, 2005; Stiles, 1975) or floral isolation (premating barrier caused by differences in morphological, visual or olfactory traits; e.g., Fulton & Hodges, 1999; Maad & Nilsson, 2004; Nilsson, 1983; Sun, Schlüter, Gross, & Schiestl, 2015). If floral trait differences between closely related taxa are maintained by selection, we should expect current divergent selection on these traits.

Adaptive divergence occurs when selection drives the evolution of traits toward different optima in different populations or species. Depending on the current trait distributions in relation to these respective optima, divergent selection can be linear in different directions (e.g., Hall & Willis, 2006) or stabilizing with different optima (e.g., Benkman, 2003). Divergent selection on flowering phenology has been documented between lowland and montane populations of *Mimulus guttatus* (Hall & Willis, 2006), between lowland and alpine populations of *Arabidopsis lyrata* (Sandring, RiihimäKi, Savolainen, & Ågren, 2007), and between diploid and tetraploid *Heuchera grossulariifolia* (Nuismer & Cunningham, 2005). Divergent selection on floral morphology has been detected in several studies, including traits that influence the efficiency of pollen transfer such as tube or spur length (Gómez, Perfectti, Bosch, & Camacho, 2009; Rymer, Johnson, & Savolainen, 2010), and traits that influence the attraction of pollinators such as corolla size (Campbell, 2003; Gómez et al., 2009) and number of inflorescences (Sandring et al., 2007). Most of these studies provide examples of divergent selection within species, and only a few studies have tested whether floral differentiation between species is maintained by divergent selection. While there was divergent selection on corolla size between two *Ipomopsis* species visited by hummingbirds and hawkmoths (Campbell, 2003), this was not the case between two *Lobelia* species specialized on hummingbirds and bumblebees, respectively (Johnston, 1991). To elucidate which traits contribute to the maintenance of species boundaries, it is necessary to study selection on floral traits that are differentiated between closely related taxa.

In this study, we quantify phenotypic selection on flowering phenology, three floral display traits and spur length in the closely related orchids *Gymnadenia conopsea* s.s. and *Gymnadenia densiflora* on the island of Öland, southern Sweden. These two species constitute an excellent system to study divergent selection on floral traits. First, the two species differ in flowering phenology and flower morphology, but also exhibit partly overlapping quantitative variation in these traits in the wild (Jersáková et al., 2010; Stark, Michalski, Babik, Winterfeld, & Durka, 2011). Second, both orchids depend on pollinators for successful fruit set, and significant pollinator‐mediated selection on flowering phenology, floral display, and spur length has been documented in *G. conopsea* s.s. (Chapurlat, Ågren, & Sletvold, 2015; Sletvold & Ågren, 2010; Sletvold, Trunschke, Wimmergren, & Ågren, 2012). Third, the pollinator communities partly differ between the two species, and on Öland, *G. conopsea* s.s. is mainly visited by nocturnal pollinators, while *G. densiflora* is mainly visited by diurnal pollinators with shorter proboscis than the nocturnal ones (Chapurlat, Anderson, Ågren, Friberg, & Sletvold, 2018; this study). Fourth, genetic studies suggest interspecific gene flow and introgression between the species, where introgression is associated with reduced fitness (Gustafsson & Lönn, 2003; Lönn, Alexandersson, & Gustafsson, 2006). Our objective is to test for divergent selection on flowering phenology and floral morphology between the two *Gymnadenia* species. On Öland, *G. conopsea* s.s. flowers earlier than *G. densiflora*, produces shorter inflorescences with fewer flowers and longer spurs, and is pollinated by species with longer proboscis (see below). If trait differences are adaptive, we expect optimal flowering to be earlier, optimal flower production and plant height to be lower and optimal spur length to be longer in *G. conopsea* s.s. than in *G. densiflora*. Given sufficient trait variation, this should be evident as directional selection of opposite sign, or stabilizing selection with different optima in the two species.

## 2 MATERIALS AND METHODS

### 2.1 Study species

*Gymnadenia conopsea* (L.) s.l. is a terrestrial orchid distributed across Eurasia (Hultén & Fries, 1986). The tuberous, nonclonal, and long‐lived perennial plant prefers calcareous soils in grazed or mown meadows and margins of marshes and fens (Øien & Moen, 2002). The *Gymnadenia conopsea* (L.) s.l. complex is highly variable with regard to morphology, scent production, flowering phenology, and habitat (Gustafsson & Lönn, 2003; Jersáková et al., 2010; Soliva & Widmer, 1999; Stark et al., 2011). The most recent classification based on genetic data recognizes two taxa within the *G. conopsea* (L.) s.l. complex: *G. conopsea* (L.) R.Br. s.s. and *G. densiflora* A. Dietr (Bateman et al., 2003; Stark et al., 2011). These two taxa were previously considered subspecies based on morphological similarity, but they do not even have a sister‐species relationship as phylogenetic analyses of the genus have shown that *G. odoratissima* is the sister species of *G. conopsea* s.s. (Bateman et al., 2003; Brandrud, Paun, Lorenz, Baar, & Hedrén, 2019; Sun et al., 2015). *Gymnadenia odoratissima* differs from the other taxa in color, floral scent, and morphology and was thus not previously included in the *G. conopsea* (L.) s.l. complex. Furthermore, variation in ploidy levels ranging from diploids to hexaploids has been reported in *G. conopsea* s.s., with diploids and tetraploids being the major cytotypes (Trávníček et al., 2012). No tetraploid *G. conopsea* s.s. has been found in Sweden, where diploids dominate, even though some triploid individuals have been identified (Stark et al., 2011; Travnicek et al., 2012). *Gymnadenia densiflora* is reported to be diploid across the European range (Marhold, Jongepierová, Krahulcová, & Kučera, 2005; Stark et al., 2011; Trávnícek et al., 2012).

**FIGURE 1:**
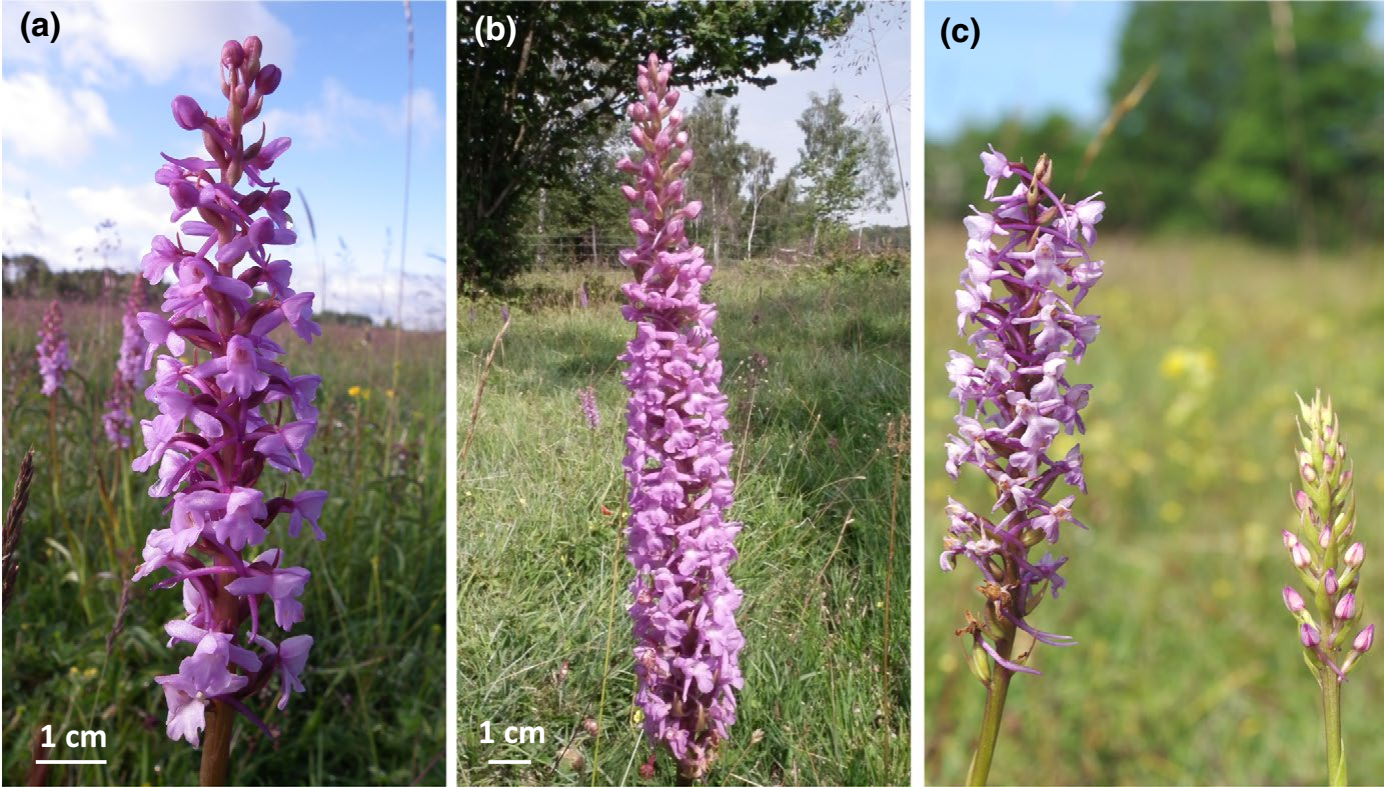
Illustration of the two study species, the fragrant orchids *Gymnadenia conopsea* s.s. (a) and *Gymnadenia densiflora* (b) that differ in plant height and floral display and particularly in flowering time (c), as shown at a site where they co‐occur: *G. conopsea* s.s. (left) has initiated fruit development while *G. densiflora* (right) is still in bud

Both species produce a single inflorescence of ca 10–100 fragrant pink flowers (Figure 1) that open sequentially from the bottom to the top of the inflorescence. Individual flowers remain open for up to a week while individual plants may flower for a month. A narrow spur contains nectar that is produced throughout anthesis (Stpiczynska & Matusiewicz, 2001). Each flower contains two pollinaria which are situated above the spur entrance. Both species are self‐compatible, but depend on pollinators for successful fruit set (Sletvold, Grindeland, Zu, & Ågren, 2012). The available literature indicates that diploid *G. conopsea* s.s. flowers earlier than *G. densiflora* (Jersáková et al., 2010) and produces shorter inflorescences with fewer flowers (Stark et al., 2011). The two species also differ in floral scent (Jersáková et al., 2010). In contrast, there is no consistent difference in spur length, as *G. conopsea* s.s. had shorter spurs than *G. densiflora* in a study conducted in the Czech Republic (Jersáková et al., 2010), while the opposite has been reported in Germany (Stark et al., 2011).

### 2.2 Study sites and pollinator communities

**FIGURE 2:**
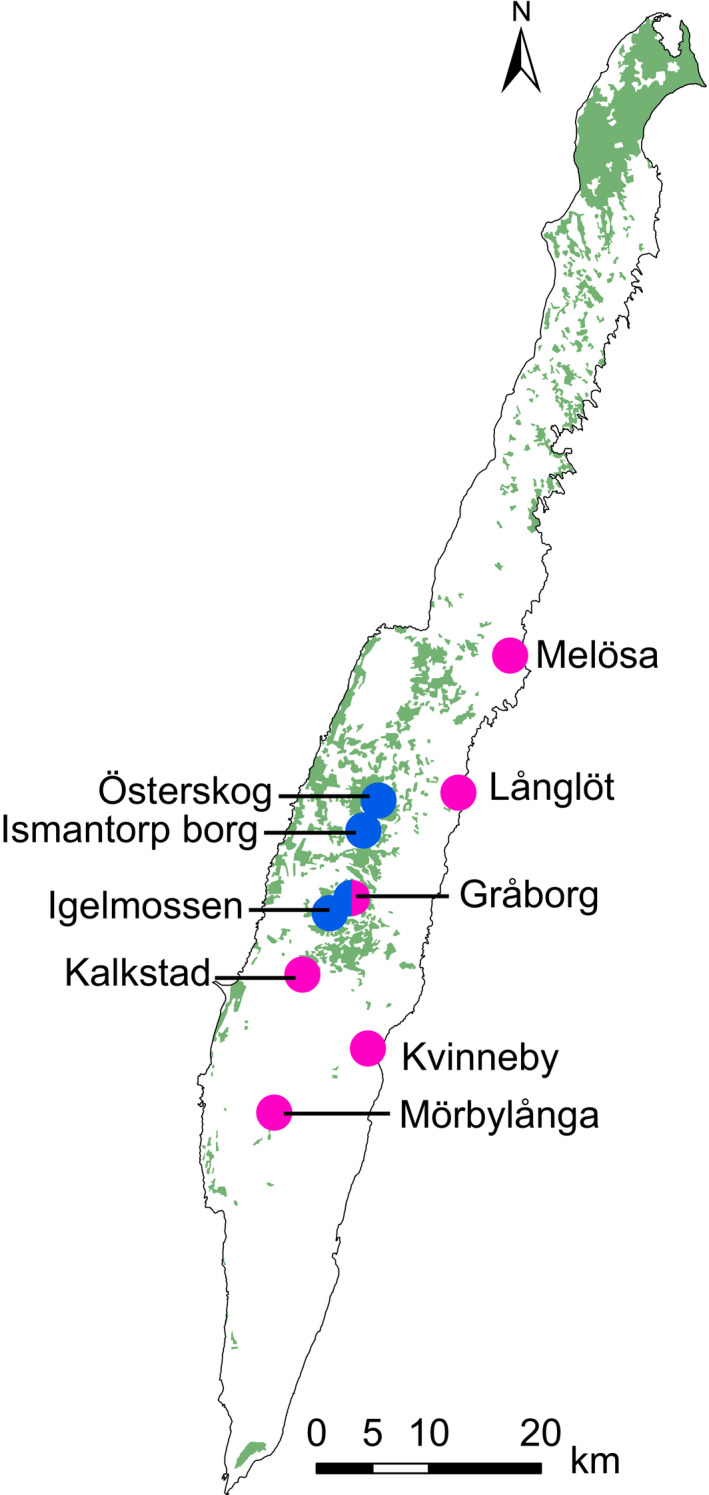
Locations of the six *Gymnadenia conopsea* s.s. and four *Gymnadenia densiflora* study populations on the island of Öland, southern Sweden. Pink symbol = *G. conopsea* s.s. population; blue symbol = *Gymnadenia densiflora* population; mixed symbol = site where both species were studied

The ten study populations are located on the calcareous island Öland, southeastern Sweden (Figure 2). All populations contained >140 flowering individuals and are separated from each other by a minimum of 2 km. The populations are located in forest meadows or open grasslands. On Öland, the two species occurs in isolation, in close proximity (20–100 m) but with slight habitat separation, as well as in truly mixed populations. Flow cytometry conducted on leaves (see below) revealed that the two species grow in sympatry (populations ≤100 m apart) at five of the sites (Gråborg, Igelmossen, Ismantorp, Kalkstad, Melösa) but, except at Gråborg, selection was quantified in only one of the species at each site.

On Öland, the two species share several nocturnal pollinators, namely *Autographa gamma*, *Deilephila porcellus,* and *Hyles gallii*, but *G. conopsea* s.s. is also pollinated by additional nocturnal Lepidopterans, such as *Cucullia umbratica* and *Agrotis exclamationis*. In contrast, diurnal pollinators differ for the two species, with *G. conopsea* s.s. being visited by diurnal Lepidopterans (*Aglais urticae*, *Zygaena minos*, *Siona lineata*) and occasionally by *Empis* flies, whereas *G. densiflora* is pollinated by a different set of diurnal Lepidopterans (including *Aglais io*, *Argynnis paphia*, *Gonopteryx rhamni*, *Issoria lathonia*, *Ochlodes sylvanus*, *Zygaena filipendula*). Pollinator catches in the study populations indicate that proboscis length of the main nocturnal pollinators on average is 7.4 mm longer than that of diurnal pollinators (means based on species means [range], 19.1 [15.8–23.6] mm vs. 11.7 [9.1–14.7] mm; Table S2). While flowers of both species are visited both diurnally and nocturnally, nocturnal visitors are more frequent than diurnal ones in populations of *G. conopsea* (mean visits per hour, 6.8 vs. 0), whereas the opposite trend is observed in populations of *G. densiflora* (0.6 vs. 1.9), based on 123 hr video recordings at night, and 68 hr at day, in two populations of each species. Nocturnal pollinators also contribute more than diurnal pollinators to reproductive success of *G. conopsea* s.s. (Chapurlat et al., 2015, 2018).

### 2.3 Measured traits for selection analysis

Plant traits and estimates of female reproductive success were recorded in summer 2012 for 120 individuals in each of the ten populations. We visited each population at least twice during the flowering period, and flowering start was recorded for each individual as the estimated day on which the first flower opened based on detailed observations in two populations that were visited daily and that indicate that three flowers open per day (data from Långlöt and Melösa, *n* = 480 plants in each population). We recorded the height of each plant as the distance from ground to topmost flower. On one of the flowers in the lower third of the inflorescence, we measured spur length (distance from corolla to spur tip) and maximum corolla width and height to the nearest 0.1 mm with digital calipers. We quantified corolla size as the product of corolla width and height and counted the number of flowers at fruit maturation.

To quantify female reproductive success, we recorded the number of fruits at maturation, and, when possible, collected three nondehisced capsules spread across the inflorescence to determine mean fruit mass for each plant. Fruit mass is positively related to number of seeds with embryos in *G. conopsea* s.s. (linear regression, *b* = 0.40, *R* ^2^ = .67, *n* = 44, each fruit sampled from a separate individual; Sletvold & Ågren, 2010). In all *G. conopsea* s.s. populations, some capsules had dehisced before fruit collection. Fruit mass is positively related to the fruit volume in *G. conopsea* s.s. (Chapurlat et al., 2015), and we used the following equation to estimate fruit mass before dehiscence from fruit volume of the dehisced capsules: fruit mass (mg) = 0.136 × fruit volume (mm^3^) + 1.65, *r* ^2^ = .87, with volume = fruit length × π × (fruit width/2)^2^. The proportion of open fruits was under 26% in all populations except Kvinneby (50%) and Gråborg (96%). For each plant, we estimated female fitness as the product of number of fruits and mean fruit mass.

### 2.4 Species identification by flow cytometry

*Gymnadenia conopsea* s.s. and *G. densiflora* are difficult to distinguish in the field because of overlapping variation in floral traits and phenology as well as variation in ploidy levels within *G. conopsea* s.s. in parts of its range (Jersáková et al., 2010; Stark et al., 2011). However, flow cytometry can reliably identify *Gymnadenia* species (Travnicek et al., 2011, 2012) because the species differ in both genome size and proportion of endoreplicated genome (about 12% smaller genome size and 28% higher proportion of endoreplicated genome in *G. densiflora*; Trávníček et al., 2012), yielding species‐ and ploidy‐specific fluorescence profiles. We therefore used flow cytometry to verify species identification based on phenology in the field in 2012 and to check for possible variation in ploidy levels in our study populations. On 14th and 15th of June 2014, we collected leaf samples from 21 to 90 individuals in each population. We sampled the whole range of phenologies present in a given population and collected more samples when there was pronounced variation in flowering phenology. We sampled leaves from at least seven plants belonging to each of three flowering time categories; “early” (*n* = 287), “intermediary” (*n* = 53), or “late” (*n* = 171), where intermediary individuals were those that began flowering during the period of overlap in flowering start (i.e., when observations were made of plants beginning to flower in *G. conopsea* as well as in *G. densiflora* populations). Leaf samples from *Gymnadenia* and from the standard *Pisum sativum* “Ctirad” were placed in 1 L plastic bags together with a moist paper towel and shipped to the Plant Cytometry Services company in The Netherlands (https://plantcytometry.com/) where they were processed within a couple of days.

Leaf samples from *Gymnadenia* plants were analyzed together with the internal standard *Pisum sativum* “Ctirad” (C = 9.09 pg) as in Travnicek et al. (2011) to allow taxa identification. Intermediary individuals were always analyzed separately. For early and late individuals, up to three leaf samples from the same phenological group and population were pooled, leading to a total of 240 flow cytometry analyses. Nuclei were stained with DAPI (4,6‐diamidino‐2‐phenylindole). We based identification on a combination of two peak ratios, following Travnicek et al. (2011; Table S1). There was no strong indication of variation in ploidy levels in our study populations, although three samples (one each from Kalkstad, Långlöt, and Ismantorp) could potentially be *G. conopsea* s.s. triploids. The correlation between phenology and taxon was high across populations: “Early” samples corresponded to *G. conopsea* s.s. individuals in 116 out of 117 analyses (99.1%), “late” samples corresponded to *G. densiflora* individuals in 69 out of 70 analyses (98.6%), whereas “intermediary” samples were mixed (53 analyses). Based on this, we checked the 2012 dataset for “intermediary individuals,” and we excluded the six latest flowering individuals at Kalkstad and the two earliest flowering individuals at Ismantorp borg because these individuals had a flowering start that clearly deviated from other plants growing at these sites. This constituted 0.0076% of the total phenotypic selection dataset (*n* = 1,056).

### 2.5 Statistical analyses

All analyses were conducted with R 3.1.3 (R Core Team, 2015). Data from four of the study populations (*G. densiflora* at Gråborg, *G. conopsea* s.s. at Kvinneby, Långlöt, and Melösa) were also included in a previous study (Chapurlat et al., 2015).

Phenotypic correlations were quantified with Pearson's correlation coefficient. To visualize the phenotypic distribution of floral traits in each population, we used the smoothing *density* function with a gaussian kernel. To determine whether floral and reproductive traits differed between species, we used the *lmer* function from the *lme4* package and specified a mixed‐effect model with species as fixed effect and population as a random factor nested within species and tested the significance of the effect of species comparing the full model and the null model without the species effect with the ANOVA function (likelihood ratio test).

Directional selection was estimated following Lande and Arnold (1983), using multiple regression analyses with relative fitness (individual female fitness divided by mean fitness) as the response variable and standardized trait values (with a mean of 0 and a variance of 1) as explanatory variables. Relative fitness and standardized trait values were calculated separately for each population. We estimated directional selection gradients (β*_i_*) from multiple regression models including only linear terms and separately for each population. We quantified nonlinear gradients (γ*_ii_*) from the quadratic terms of the full regression models (Lande & Arnold, 1983). The reported γ*_ii_* are obtained by doubling the coefficients extracted from the regression model to represent quadratic selection gradients (Stinchcombe, Agrawal, Hohenlohe, Arnold, & Blows, 2008). Multicollinearity was assessed by inspection of variance inflation factors (VIF), which in no case exceeded 2.3 for the models including only linear terms and 9.7 for the full models, indicating that the level of collinearity was not problematic (Quinn & Keough, 2002).

Phenotypic selection studies cannot distinguish the causal effects of focal traits from potential environmentally induced covariances between traits and fitness unless trait expression is manipulated (Mauricio & Mojonnier, 1997; Rausher, 1992). This is likely to be a problem mainly for size‐related traits, and the best approach to deal with this if you cannot use genotypic selection is to include measures of overall plant size in the model. We included both plant height and number of flowers in our phenotypic selection models.

To test for divergent linear selection, we conducted for each floral trait a one‐sided Welch *t* test on the linear selection gradients, with the alternative hypothesis being that selection gradients are greater in the species with the largest mean trait value. We examined whether there was stabilizing selection (presence of an intermediate optimum) graphically by the use of added‐variable plots.

## 3 RESULTS

### 3.1 Differences in floral traits and reproductive performance between the two species

**FIGURE 3:**
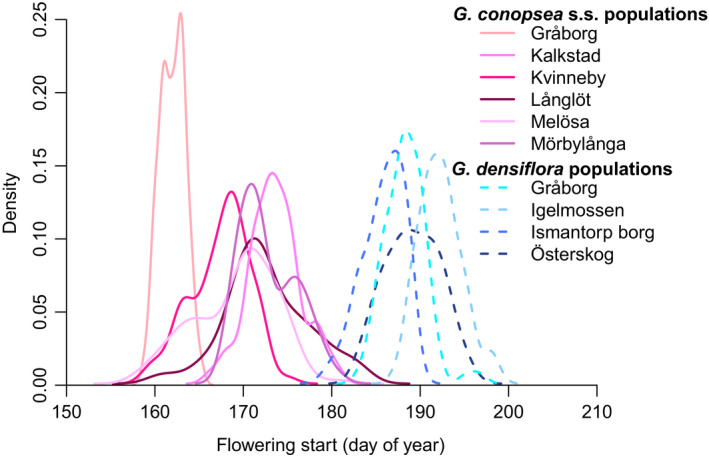
Phenological density curves based on estimated flowering start for each *Gymnadenia conopsea* s.s. (solid pink lines) and *Gymnadenia densiflora* (blue dashed lines) study population in 2012

Flowering start and floral display differed between the two species (Table 1). On average, *Gymnadenia conopsea* s.s. individuals flowered earlier (Figure 3), were shorter, produced fewer and smaller flowers but had longer spurs than *G. densiflora* individuals, although the difference in spur length was only marginally significant (Table 1; Figure S1). The observed phenotypic distributions overlapped between species, ranging from a small overlap for flowering start (Figure 3) to a large overlap for the morphological traits (Figure S1). Floral traits were moderately positively correlated within each population, except flowering date, which tended to be negatively correlated with the other traits (Table S3). Number of fruits and fruit mass differed significantly between the two species (Table 1). *Gymnadenia conopsea* s.s. individuals produced fewer but heavier fruits than did *G. densiflora* individuals, which led to marginally significant higher average female fitness for *G. densiflora*.

**TABLE 1:** Plant traits and reproductive performance (mean ± *SD*) for plants in the *Gymnadenia conopsea* s.s. and *Gymnadenia densiflora* populations in 2012. The values at the species level are the estimates ± *SE* extracted from the mixed‐effect model including population as random effect and species as fixed factor. The species effect was tested with a likelihood ratio test (LRT): *p* < .05 are indicated in bold. Populations are ordered by mean flowering start

Species	Population (sample size)	Floral traits					Reproductive performance		
		Flowering start (day of year)	Plant height (cm)	Number of flowers	Corolla size (mm^2^)	Spur length (mm)	Number of fruits	Fruit mass (mg)	Female fitness
*G. conopsea* s.s.	Gråborg (*n* = 86)	162 ± 1.4	24.3 ± 4.8	30.6 ± 9.8	95.2 ± 19	14.0 ± 1.8	24.6 ± 11	13.6 ± 3.9	355 ± 228
	Kvinneby (*n* = 83)	167 ± 3.3	24.4 ± 5.1	32.6 ± 10	101.3 ± 28	15.1 ± 1.9	25.5 ± 10	9.8 ± 3.6	264 ± 176
	Melösa (*n* = 116)	169 ± 4.6	20.8 ± 5.2	34.3 ± 12	113.2 ± 26	14.8 ± 1.8	23.5 ± 14	11.4 ± 4.1	293 ± 264
	Långlöt (*n* = 105)	173 ± 4.8	26.7 ± 6.2	31.2 ± 11	100.8 ± 26	14.7 ± 1.8	24.1 ± 13	10.2 ± 3.8	262 ± 180
	Mörbylånga (*n* = 107)	173 ± 3.2	28.6 ± 6.0	36.9 ± 11	84.5 ± 21	15.5 ± 2.0	30.9 ± 12	9.7 ± 3.3	313 ± 181
	Kalkstad (*n* = 98)	174 ± 2.9	30.6 ± 6.8	29.4 ± 10	88.3 ± 20	16.1 ± 2.0	24.3 ± 11	10.6 ± 3.5	261 ± 148
	**Species mean ± *SE***	**170 ± 1.4**	**25.9 ± 1.4**	**32.5 ± 0.81**	**97.2 ± 3.7**	**15.0 ± 0.21**	**25.5 ± 0.89**	**10.9 ± 0.50**	**291 ± 18**
*G. densiflora*	Ismantorp borg (*n* = 115)	186 ± 2.4	33.4 ± 6.5	41.3 ± 12	126.9 ± 24	14.5 ± 1.6	37.6 ± 12	9.8 ± 2.7	380 ± 191
	Gråborg (*n* = 114)	188 ± 2.3	28.2 ± 7.4	41.2 ± 13	103.5 ± 19	14.1 ± 1.3	33.6 ± 14	7.1 ± 2.1	252 ± 146
	Österskog (*n* = 108)	189 ± 3.1	37.1 ± 8.7	42.4 ± 15	111.8 ± 22	14.6 ± 1.4	36.6 ± 15	9.2 ± 3.6	366 ± 283
	Igelmossen (*n* = 116)	192 ± 2.4	38.5 ± 8.2	42.5 ± 17	117.5 ± 25	14.2 ± 1.6	36.6 ± 17	9.7 ± 3.1	377 ± 251
	**Species mean ± *SE***	**189 ± 2.2**	**34.3 ± 2.3**	**41.8 ± 1.3**	**114.9 ± 5.9**	**14.4 ± 0.33**	**36.1 ± 1.4**	**9.0 ± 0.79**	**344 ± 28**
*p* (effect of species)		**<.0001**	**.0034**	**<.0001**	**.011**	.067	**<.0001**	**.030**	.079

### 3.2 Differences in selection in the two *Gymnadenia* species

**FIGURE 4:**
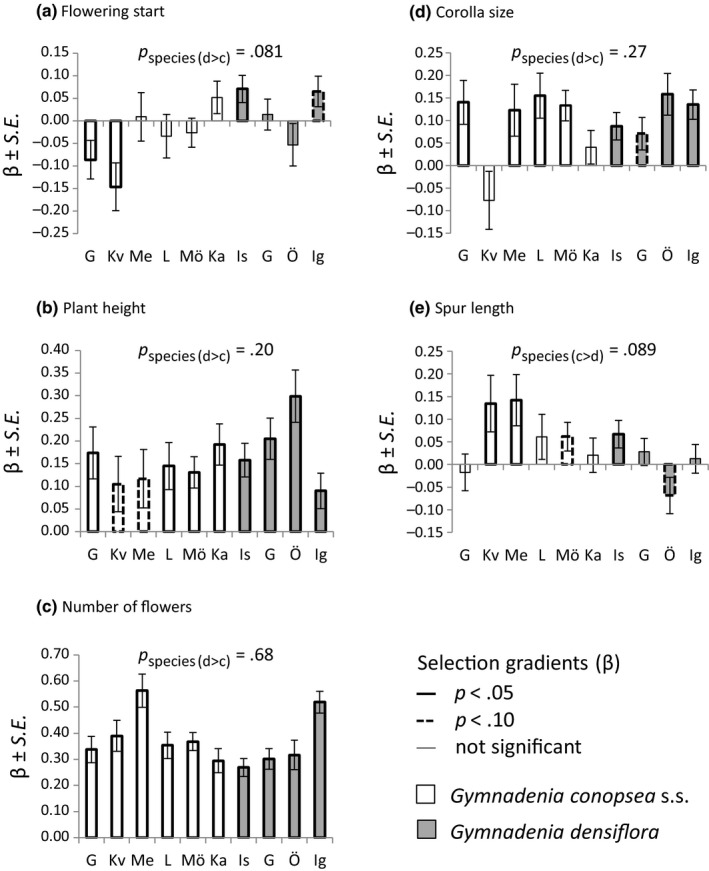
Linear selection gradients (β) ± *SE* for five floral traits (panels a–e) in the six populations of *Gymnadenia conopsea* s.s. (white bars) and four populations of *Gymnadenia densiflora* (gray bars) in 2012. The names of each population are abbreviated on the *x* axis as follows: G, Gråborg; Ig, Igelmossen; Is, Ismantorp; Ka, Kalkstad; Kv, Kvinneby; L, Långlöt; Me, Melösa; Mö, Mörbylånga; Ö, Österskog. Significant (*p* < .05) and marginally significant (*p* < .10) gradients are indicated by a thicker solid and dashed outline, respectively. Populations are ordered by mean flowering start. The *p*‐value associated with the one‐sided Welch *t* test testing for differences in selection gradients between the two species is indicated above each bar plot, with the tested alternative hypothesis indicated between parenthesis (c, conopsea; d, densiflora; > =“greater than”)

In both species, there was significant directional selection on all floral traits included in the analysis, but only flowering start tended to experience divergent selection between the two species (Figure 4; Table S4). There was selection for earlier flowering in two *G. conopsea* s.s. populations and for later flowering in one *G. densiflora* population (Figure 4; Table S4). In addition, selection for longer spurs tended to be stronger in *G. conopsea* s.s. than in *G. densiflora*, but selection on display traits did not differ between species (Figure 4; Table S4).

There was no indication of divergent stabilizing selection. Only two quadratic gradients were statistically significant; one positive for number of flowers in Kalkstad, and one negative for spur length in Ismantorp (Table S5). However, added‐variable plots revealed that the negative quadratic selection gradient for spur length reflected curvature but no intermediate optimum.

## 4 DISCUSSION

In this study, we tested the hypothesis that floral divergence between *G. conopsea* s.s. and *G. densiflora* is mirrored by current divergent selection on flowering phenology and morphology. Partially consistent with this hypothesis, we documented divergent linear selection on flowering time between some populations. In contrast, there was no indication of divergent selection on morphological traits between the two species.

One of the main differences between the two studied *Gymnadenia* species is flowering phenology. The selection patterns documented in this study are partially consistent with this differentiation, as selection for earlier flowering was detected in two of the early‐flowering *G. conopsea* s.s. populations, and selection for later flowering in one of the late‐flowering *G. densiflora* populations. Genetic surveys in southern Sweden have found some evidence of gene flow and introgression between *G. conopsea* s.s. and *G. densiflora*, where introgression into *G. densiflora* was associated with reduced fitness (Gustafsson & Lönn, 2003; Lönn et al., 2006). Interspecific pollen deposition during the overlapping flowering period may thus be costly and could potentially cause divergent selection, as has been hypothesized for diploid and tetraploid *Heuchera* (Nuismer & Cunningham, 2005). Indeed, both species grow in sympatry in three of the four populations where we detected significant or marginally significant selection on phenology. However, in this scenario, the strongest selection gradients on phenology should occur in the populations with more intermediate phenologies, which was not the case. It is thus unclear if interference contributes to the observed divergent selection. Quantifying rates of interspecific pollen transfer in natural populations together with experimental crosses between the two *Gymnadenia* species would be necessary to test this hypothesis. Alternatively, pollinators or abiotic agents could cause the selection on phenology observed in our study populations, as has been shown in this and other plant species (Elzinga et al., 2007; Pilson, 2000; Sandring & Ågren, 2009; Sletvold, Grindeland, & Ågren, 2010; Sletvold, Moritz, & Ågren, 2015). In four of the included study populations, spatial variation in net selection on flowering start is partly explained by variation in pollinator‐mediated selection (Chapurlat et al., 2015). The divergent selection observed between *G. conopsea* s.s. and *G. densiflora* could thus be caused by temporal variation in pollinator communities throughout the flowering season. However, some of the net selection on flowering start is nonpollinator mediated in the Kvinneby population, suggesting that abiotic factors could also contribute to the selection gradients (Chapurlat et al., 2015). Phenological isolation between two plant taxa is the earliest premating barrier possible and has the greatest potential for reproductive isolation (Widmer, Lexer, & Cozzolino, 2009), and our results suggest that divergent natural selection should reinforce this barrier between the two *Gymnadenia* species.

The strength and direction of linear selection on spur length, a trait influencing the efficiency of pollination (Boberg & Ågren, 2009; Ellis & Johnson, 2010; Nilsson, 1988; Sletvold & Ågren, 2011; Trunschke et al., 2019), varied among populations, but there was little evidence of divergent selection between the two species. Overall, selection on spur length tended to be stronger in the longer‐spurred species, *G. conopsea* s.s., with significant selection for longer spurs in two of the six populations. In the shorter‐spurred *G. densiflora*, there was selection for longer spurs in one population. Selection on spur length in *G. conopsea* s.s. and other species has repeatedly been shown to be mediated by pollinators (Chapurlat et al., 2015; Sletvold & Ågren, 2014; Sletvold et al., 2010; Trunschke, Sletvold, & Ågren, 2017). The pollinator communities differ partly between our study populations, and in particular between *G. conopsea* s.s. and *G. densiflora*, which could explain variation in selection patterns on spur length. Available data indicate that on Öland, *G. densiflora* is visited by pollinators that have shorter probosces than pollinators visiting *G. conopsea* s.s. (Table S2). However, it is unclear whether the relatively small difference in spur length contributes to floral isolation between the two study species. Previous studies that suggest floral isolation due to spur length differences report considerably larger differences in spur lengths between taxa (e.g., Anderson, Alexandersson, & Johnson, 2010; Fulton & Hodges, 1999; Nilsson, 1983, 1988; Sun et al., 2015). Furthermore, reports on mean spur length in the two species indicate that the direction of difference varies throughout their range (this study, Jersáková et al., 2010; Stark et al., 2011). Studies that characterize differences in pollinator communities and test for floral isolation between the two *Gymnadenia* species in several parts of their range could help elucidate whether local differences in spur length are adaptive.

There was no evidence of divergent selection on floral display traits, that is, plant height, number of flowers and corolla size, in spite of significant differences in these traits between the two *Gymnadenia* species, suggesting this differentiation is nonadaptive. Rather, the differences in display traits may in part represent plastic responses to habitat differences between species. *Gymnadenia densiflora*, which on average produces larger floral displays than *G. conopsea* s.s., grows in more moist conditions (Gustafsson & Lönn, 2003), which could favor growth. Differentiation in these traits may also be caused by pleiotropic effects if they are genetically correlated with other floral trait(s) that have been subject to divergent selection. Because *G. densiflora* begins to flower later, it has more time to gather resources before flowering and may therefore be able to produce larger floral displays (cf. Elzinga et al., 2007; Mitchell‐Olds, 1996). Although difficult to conduct in orchids, common‐garden experiments with half‐sib crossings would be the ideal way to test for genetic differences and genetic correlations among traits in the two species.

Both studied species are long‐lived perennials, and potential trade‐offs across the life cycle may cause selection estimated via a single fitness component to deviate from estimates via lifetime fitness (e.g., Gómez, 2008). Field experiments in *G. conopsea* populations in Norway demonstrate that maximizing fruit production via supplemental hand‐pollination is associated with significant short‐term costs in terms of reduced survival, flowering probability, and fruit production the next year, compared to individuals with natural pollination and fruit production (Sletvold & Ågren, 2011b, 2015). However, using a combination of experimental and long‐term demographic data, Tye, Dahlgren and Sletvold (2020) showed that such costs do not carry over to later years and are too weak to counteract the advantage of high seed production in the first year. This suggests a minor role of conflicting selection via other fitness components, and a substantial correlation between seed production in a single season and lifetime female fitness. Ideally, effects on male fitness should also be considered, but because pollen removal is often a poor predictor of pollen export (Johnson, Neal, & Harder, 2005) or siring success (Snow & Lewis, 1993), paternity analyses would be required to reliably quantify selection through male function.

While many studies have examined whether spatial variation in selection on floral traits can explain differentiation of these traits within species (Chapurlat et al., 2015; Gómez et al., 2008, 2009; Gross, Sun, & Schiestl, 2016; Hall & Willis, 2006; Sandring et al., 2007; Schueller, 2007), our study is among the first to test whether variation in selection on floral traits can explain the maintenance of floral trait divergence between closely related species (but see Campbell, 2003; Joffard, 2017; Johnston, 1991). Our results indicate that divergent selection contributes to the marked phenological differentiation between *Gymnadenia conopsea* s.s. and *Gymnadenia densiflora*, but also show that current selection patterns do not mirror morphological floral divergence between the two species. This suggests that nonadaptive processes such as genetic drift or pleiotropic constraints may play a role in the floral trait differentiation between the two species, or that selection has driven this differentiation historically but is not strong any longer (Harder & Johnson, 2009). Further investigations are needed to fully understand whether floral differentiation between *G. conopsea* s.s. and *G. densiflora* is adaptive, and the extent to which phenological and floral isolation act as reproductive barriers between the two species. Phenological isolation between two plant taxa has a substantial potential for reproductive isolation (Widmer et al., 2009), and divergent selection on flowering time reported here and in other studies (Hall & Willis, 2006; Nuismer & Cunningham, 2005; Sandring et al., 2007) can thus greatly influence reproductive isolation and differentiation.

## CONFLICT OF INTEREST

The authors declare that they have no conflict of interest.

## AUTHOR CONTRIBUTION

**Elodie Chapurlat:** Conceptualization (equal); data curation (lead); formal analysis (lead); investigation (equal); methodology (equal); visualization (equal); writing – original draft (lead); writing – review and editing (equal). **Iris Le Roncé:** Formal analysis (equal); writing – original draft (supporting); writing – review and editing (supporting). **Jon Agren:** Conceptualization (equal); methodology (equal); supervision (supporting); writing – review and editing (equal). **Nina Sletvold:** Conceptualization (equal); data curation (supporting); formal analysis (supporting); funding acquisition (lead); methodology (equal); supervision (lead); writing – review and editing (equal).

## Supporting information

Supplementary Material

## Data Availability

Data are archived with Dryad: https://doi.org/10.5061/dryad.b2rbnzsbd
