# Supplementary material for: Divergent selection on flowering phenology but not on floral morphology between two closely related orchids
Source: Ecol Evol. 2020 May 6;10(12):5737–47. doi: 10.1002/ece3.6312 (PMC7319237; doi:10.1002/ece3.6312)
Supplement: Supplementary file 1 — Supplementary Material [file ECE3-10-5737-s001.docx]

## Supplementary material for: Divergent selection on flowering phenology but not on floral morphology between two closely related orchids

Elodie Chapurlat, Iris Le Roncé, Jon Ågren, Nina Sletvold

**Figure S1**. The distribution of plant traits within each *Gymnadenia conopsea* s.s. and *G. densiflora* study populations.

**Table S1**. Range of peak ratios observed in the flow cytometry data used for identification of *Gymnadenia conopsea* s.s. and *G. densiflora*.

**Table S2.** Pollinators caught in populations of *Gymnadenia conopsea* and *G. densiflora*.

### Table S3. Phenotypic correlations among traits within the six *Gymnadenia conopsea* s.s. and four *G . densiflora* study populations.

### Table S4. Phenotypic linear selection gradients in the *Gymnadenia conopsea* s.s. and *G. densiflora* populations.

**Table S5**. Quadratic selection gradients in the *Gymnadenia conopsea* s.s. and *G. densiflora* populations.


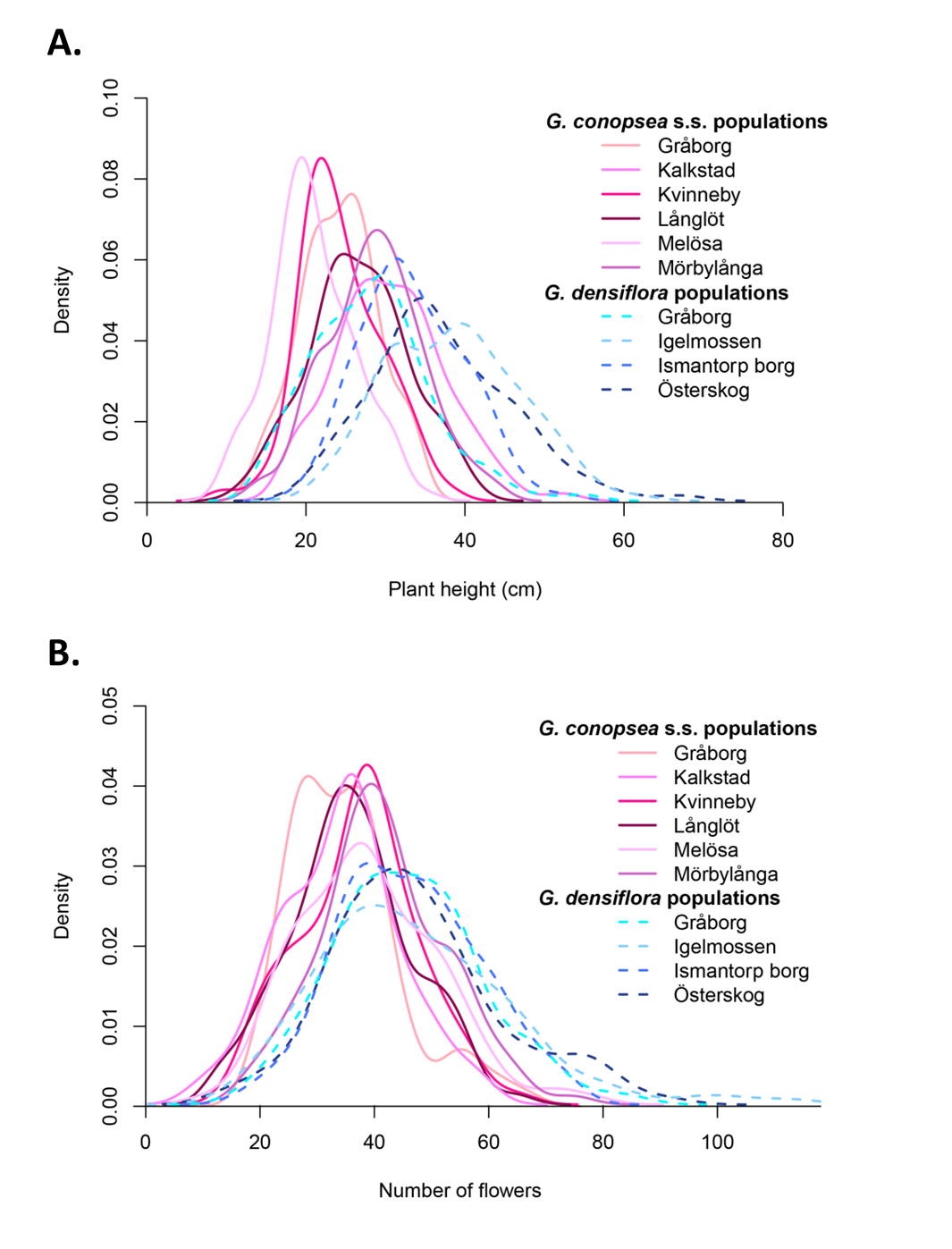


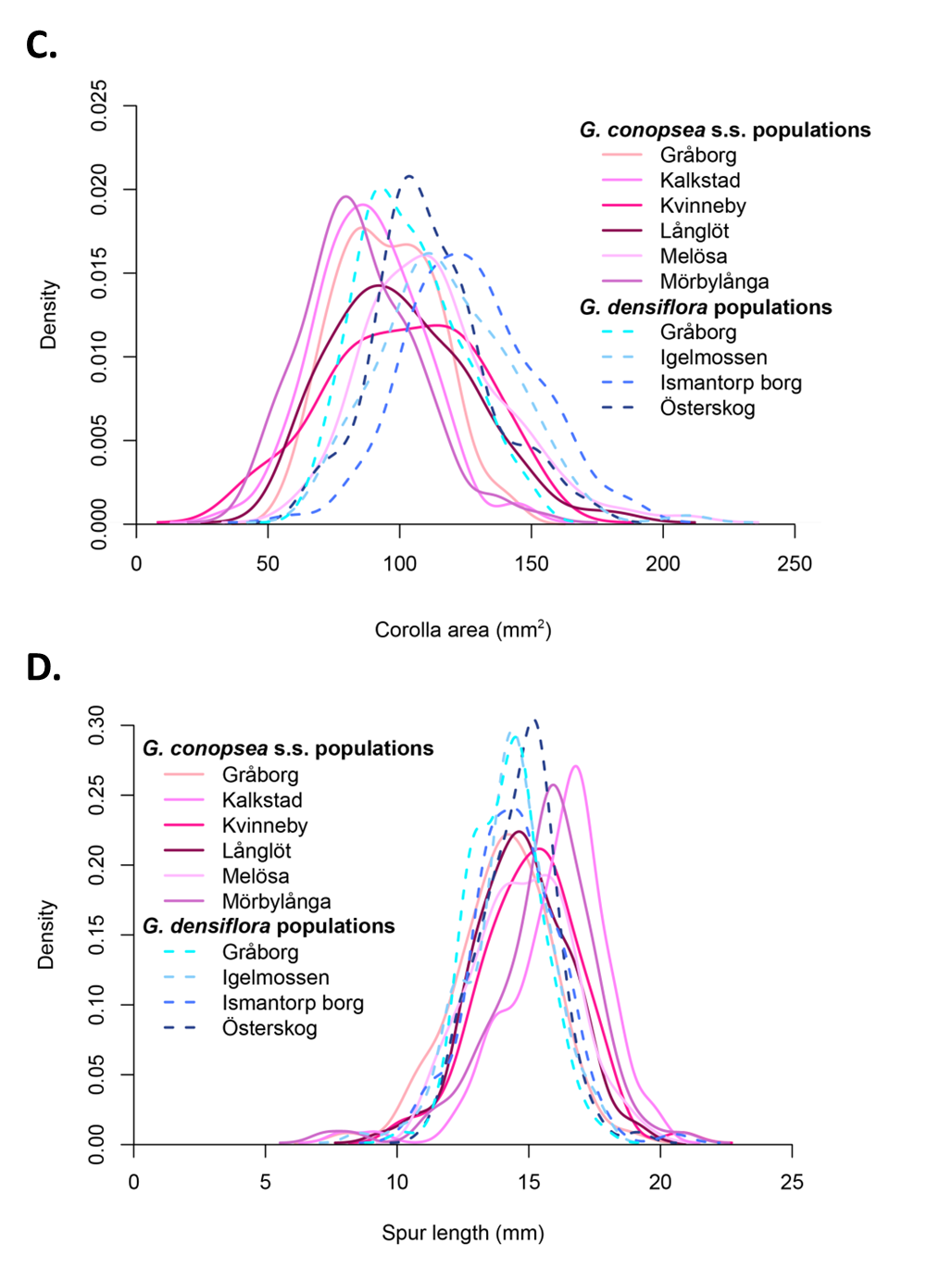


**Figure S1**. Density curves representing the distribution of plant height (**A**), number of flowers (**B**), corolla area (**C**) and spur length (**D**) within each *Gymnadenia conopsea* s.s. (solid pink lines) and *Gymnadenia densiflora* (blue dashed lines) study populations in 2012.

**Table S1**. Range of peak ratios observed in the flow cytometry data used for identification of *Gymnadenia conopsea* s.s. and *G. densiflora* in this study, with reference values from Travnicek *et al.*, 2010 indicated between brackets. 2C denotes the DNA content of the *2n* nucleus. P denotes the DNA content of the replicated portion of the *2n* nucleus resulting from the progressively partial endoreplication occurring in *Gymnadenia* and other orchids (Travnicek *et al.*, 2010). P1 is the first peak scored in the flow cytometry analysis.

|  | **Peak ratios used for identification when P1 = 2C** | | **Peak ratios used for identification when P1 = 2C+P** | |
| --- | --- | --- | --- | --- |
| **Taxon** | 2C/*Pisum sativum* | 2C + P/2C | 2C + P/*Pisum sativum* | 2C + 3P/2C + P |
| *G. conopsea* s.s. | 0.86-0.90 (0.84) | 1.55-1.63 (1.59) | 1.35-1.45 (1.33) | 1.68-1.76 (1.74) |
| *G. densiflora* | 0.77-0.82 (0.75) | 1.70-1.78 (1.75) | 1.33-1.43 (1.30) | 1.78-1.85 (1.86) |

**Table S2.** Species with pollinia attached caught in populations of *Gymnadenia conopsea* and *G. densiflora* in June-July 2016. The number of specimens of each species is given (Nb), and the mean (± SD) length of the proboscis.

| **Species** | **Period** | **G. conopsea** | **G. densiflora** | **Nb** | **Proboscis (mm)** |
| --- | --- | --- | --- | --- | --- |
| *Aglais io* | Day |  | X | 2 | 13.5 |
| *Argynnis paphia* | Day |  | X | 20 | 12.4 ± 1.1 |
| *Gonopteryx rhamni* | Day |  | X | 36 | 14.9 ± 1.3 |
| *Issoria lathonia* | Day |  | X | 1 | 11.0 |
| *Ochlodes sylvanus* | Day |  | X | 1 | NA |
| *Zygaena filipendula* | Day |  | X | 24 | 9.1 ± 1.5 |
| *Zygaena minos* | Day | X |  | 21 | 9.4 ± 0.7 |
| *Autographa gamma* | Night | X | X | 28 | 15.8 ± 0.9 |
| *Cucullia umbratica* | Night | X |  | 2 | 19 ± 1.4 |
| *Deilephila porcellus* | Night | X | X | 28 | 17.9 ± 1.2 |
| *Hyles gallii* | Night | X | X | 5 | 23.6 ± 1.8 |

### Table S3. Phenotypic correlations (Pearson’s coefficient) among traits within the six *Gymnadenia conopsea* s.s. and four *G . densiflora* study populations. (*) P < 0.1, * P < 0.05, ** P < 0.01, *** P < 0.001.

| *Gymnadenia conopsea* s.s | | | | |  | *Gymnadenia densiflora* | | | | |
| --- | --- | --- | --- | --- | --- | --- | --- | --- | --- | --- |
| **Gråborg (n=86)** | | | | |  | **Gråborg (n=114)** | | | | |
| Trait | Flowering start | Plant height | Corolla size | Number of flowers |  | Trait | Flowering start | Plant height | Corolla size | Number of flowers |
| Plant height | -0.13 |  |  |  |  | Plant height | -0.39 *** |  |  |  |
| Corolla size | 0.34 ** | 0.47 *** |  |  |  | Corolla size | -0.11 | 0.52 *** |  |  |
| Number of flowers | -0.14 | 0.66 *** | 0.28 ** |  |  | Number of flowers | -0.34 *** | 0.62 *** | 0.27 ** |  |
| Spur length | 0.038 | 0.28 ** | 0.26 * | 0.20 (*) |  | Spur length | -0.045 | 0.052 | 0.12 | -0.039 |
| **Kalkstad (n=98)** | | | | |  | **Igelmossen (n=116)** | | | | |
| Plant height | -0.043 |  |  |  |  | Plant height | -0.22 * |  |  |  |
| Corolla size | 0.10 | 0.32 ** |  |  |  | Corolla size | -0.38 *** | 0.16 (*) |  |  |
| Number of flowers | -0.16 | 0.60 *** | 0.22 * |  |  | Number of flowers | -0.41 *** | 0.68 *** | 0.17 (*) |  |
| Spur length | -0.019 | 0.29 ** | 0.027 | 0.36 ** |  | Spur length | -0.32 *** | 0.19 * | 0.42 *** | 0.15 |
| **Kvinneby (n=83)** | | | | |  | **Ismantorp borg (n=115)** | | | | |
| Plant height | -0.042 |  |  |  |  | Plant height | -0.12 |  |  |  |
| Corolla size | -0.12 | 0.32 ** |  |  |  | Corolla size | -0.31 *** | 0.34 *** |  |  |
| Number of flowers | -0.20 (*) | 0.43 *** | 0.078 |  |  | Number of flowers | -0.11 | 0.57 *** | 0.14 |  |
| Spur length | 0.046 | 0.19 (*) | 0.53 *** | -0.095 |  | Spur length | -0.28 ** | 0.26 ** | 0.15 | -0.043 |
| **Långlöt (n=105)** | | | | |  | **Österskog (n=108)** | | | | |
| Plant height | 0.1 |  |  |  |  | Plant height | -0.39 *** |  |  |  |
| Corolla size | -0.25 ** | 0.22 * |  |  |  | Corolla size | 0.0040 | 0.49 *** |  |  |
| Number of flowers | -0.22 * | 0.37 *** | 0.26 ** |  |  | Number of flowers | -0.50 *** | 0.67 *** | 0.31 ** |  |
| Spur length | 0.015 | 0.35 *** | 0.33 *** | 0.18 (*) |  | Spur length | -0.19 * | 0.083 | 0.13 | 0.0079 |
| **Melösa (n=116)** | | | | |  |  |  |  |  |  |
| Plant height | 0.049 |  |  |  |  |  |  |  |  |  |
| Corolla size | 0.085 | 0.33 *** |  |  |  |  |  |  |  |  |
| Number of flowers | 0.26 ** | 0.53 *** | 0.18 * |  |  |  |  |  |  |  |
| Spur length | 0.15 | 0.28 *** | 0.38 *** | 0.17 (*) |  |  |  |  |  |  |
| **Mörbylånga (n=107)** | | | | |  |  |  |  |  |  |
| Plant height | 0.16 (*) |  |  |  |  |  |  |  |  |  |
| Corolla size | 0.42 *** | 0.20 * |  |  |  |  |  |  |  |  |
| Number of flowers | -0.039 | 0.50 *** | 0.037 |  |  |  |  |  |  |  |
| Spur length | 0.13 | 0.23 * | 0.35 *** | 0.19 (*) |  |  |  |  |  |  |

### Table S4. Phenotypic linear selection gradients, β_i_ ± SE, with associated *P*-values in parentheses in the six *Gymnadenia conopsea* s.s. and four *G. densiflora* populations, obtained by multiple regressions including only linear terms. Populations are ordered by mean flowering start.

| **Species** | **Population** | **Flowering start** | **Plant height** | **Number of flowers** | **Corolla size** | **Spur length** |
| --- | --- | --- | --- | --- | --- | --- |
| *G. conopsea* | Gråborg | **-0.086 ± 0.04** (<0.0001) | **0.17 ± 0.06** (0.049) | **0.34 ± 0.05** (0.0032) | **0.14 ± 0.05** (<0.0001) | -0.017 ± 0.04 (0.67) |
|  | Kvinneby | **-0.15 ± 0.05** (0.0078) | 0.11 ± 0.06 (0.088) | **0.39 ± 0.06** (<0.0001) | -0.077 ± 0.06 (0.23) | **0.13 ± 0.06** (0.035) |
|  | Melösa | 0.0090 ± 0.05 (0.87) | 0.12 ± 0.06 (0.072) | **0.56 ± 0.06** (<0.0001) | **0.12 ± 0.06** (0.035) | **0.14 ± 0.06** (0.014) |
|  | Långlöt | -0.034 ± 0.05 (0.48) | **0.14 ± 0.05** (0.0060) | **0.35 ± 0.05** (<0.0001) | **0.15 ± 0.05** (0.0026) | 0.061 ± 0.05 (0.22) |
|  | Mörbylånga | -0.026 ± 0.03 (0.43) | **0.13 ± 0.03** (0.00027) | **0.37 ± 0.03** (<0.0001) | **0.13 ± 0.03** (0.00018) | 0.062 ± 0.03 (0.054) |
|  | Kalkstad | 0.052 ± 0.04 (0.15) | **0.19 ± 0.05** (<0.0001) | **0.30 ± 0.05** (<0.0001) | 0.04 ± 0.04 (0.28) | 0.021 ± 0.04 (0.59) |
| *G. densiflora* | Ismantorp borg | **0.071 ± 0.03** (0.020) | **0.16 ± 0.04** (<0.0001) | **0.27 ± 0.03** (<0.0001) | **0.087 ± 0.03** (0.0050) | **0.067 ± 0.03** (0.029) |
|  | Gråborg | 0.014 ± 0.03 (0.67) | **0.20 ± 0.05** (<0.0001) | **0.30 ± 0.04** (<0.0001) | 0.071 ± 0.04 (0.053) | 0.028 ± 0.03 (0.37) |
|  | Österskog | -0.053 ± 0.05 (0.27) | **0.30 ± 0.06** (<0.0001) | **0.32 ± 0.06** (<0.0001) | **0.16 ± 0.05** (0.00093) | -0.068 ± 0.04 (0.092) |
|  | Igelmossen | 0.065 ± 0.03 (0.056) | **0.090 ± 0.04** (0.022) | **0.52 ± 0.04** (<0.0001) | **0.14 ± 0.03** (<0.0001) | 0.013 ± 0.03 (0.69) |
| Species difference  (*P* from one-sided exact permutation t-test) | | 0.081 | 0.20 | 0.68 | 0.27 | 0.089 |

**Table S5**. Quadratic selection gradients (γ_ii_ ± SE), with associated P-values in parentheses in the six *Gymnadenia conopsea* s.s. and four *G. densiflora* populations. Populations are ordered by mean flowering start. Significant gradients are indicated in bold.

| **Species** | **Population** | **Flowering start** | **Plant height** | **Number of flowers** | **Corolla size** | **Spur length** |
| --- | --- | --- | --- | --- | --- | --- |
| *Gymnadenia conopsea* s.s. | Gråborg | -0.018 ± 0.10  (0.86) | 0.080 ± 0.14  (0.57) | 0.036 ± 0.08  (0.65) | 0.16 ± 0.10  (0.092) | -0.13 ± 0.06  (0.051) |
|  | Kvinneby | -0.018 ± 0.10  (0.85) | -0.12 ± 0.10  (0.24) | 0.20 ± 0.10  (0.053) | -0.022 ± 0.13  (0.87) | -0.094 ± 0.12  (0.44) |
|  | Melösa | 0.12 ± 0.08  (0.12) | -0.13 ± 0.12  (0.25) | -0.036 ± 0.10  (0.72) | 0.010 ± 0.07  (0.89) | -0.036 ± 0.13  (0.69) |
|  | Långlöt | -0.12 ± 0.06  (0.061) | -0.090 ± 0.10  (0.35) | 0.022 ± 0.09  (0.80) | 0.14 ± 0.09  (0.15) | -0.087 ± 0.07  (0.25) |
|  | Mörbylånga | -0.11 ± 0.07  (0.11) | -0.092 ± 0.08  (0.24) | 0.013 ± 0.06  (0.84) | -0.00098 ± 0.06 (0.99) | -0.037 ± 0.037  (0.32) |
|  | Kalkstad | 0.13 ± 0.07  (0.069) | 0.051 ± 0.13  (0.69) | **0.24 ± 0.11**  (0.027) | -0.014 ± 0.06  (0.80) | -0.042 ± 0.07  (0.53) |
| *Gymnadenia densiflora* | Ismantorp borg | -0.021 ± 0.06  (0.71) | 0.012 ± 0.07  (0.87) | -0.018 ± 0.06  (0.78) | 0.037 ± 0.05  (0.46) | 0.018 ± 0.05  (0.70) |
|  | Gråborg | -0.012 ± 0.04  (0.78) | 0.090 ± 0.10  (0.34) | -0.026 ± 0.07  (0.69) | 0.022 ± 0.08  (0.77) | 0.028 ± 0.05  (0.57) |
|  | Österskog | -0.10 ± 0.08  (0.19) | 0.018 ± 0.11  (0.87) | 0.058 ± 0.10  (0.55) | -0.078 ± 0.07  (0.23) | -0.026 ± 0.05  (0.58) |
|  | Igelmossen | -0.0086 ± 0.07  (0.91) | 0.062 ± 0.09  (0.51) | 0.11 ± 0.07  (0.12) | 0.0044 ± 0.06  (0.94) | **-0.11 ± 0.05**  (0.035) |
